# Sirtuins as Therapeutic Targets for Treating Cancer, Metabolic Diseases, and Neurodegenerative Diseases

**DOI:** 10.3390/ph18111723

**Published:** 2025-11-13

**Authors:** Maxwell Akantibila, Valerie J. Carabetta

**Affiliations:** 1 Department of Biomedical Sciences, Cooper Medical School of Rowan University, Camden, NJ 08103, USA; akanti18@students.rowan.edu; 2 Department of Chemistry and Biochemistry, Rowan University, Glassboro, NJ 08028, USA

**Keywords:** sirtuins, cancer, Alzheimer’s diseases, Huntington’s disease, SIRT, Parkinson’s disease, HDAC, deacetylase

## Abstract

Sirtuins are NAD^+^-dependent enzymes that are conserved in all domains of life, including mammals, metazoans, plasmodia, yeast, bacteria, and archaea. In humans, there are seven isoforms (SIRT1 to 7), and they function in cellular homeostasis, aging, DNA repair, survival, metabolism, and stress responses. Recent advances highlight the diverse functions of sirtuins in the pathogenesis and progression of cancer, metabolic diseases, and neurodegenerative diseases, including Alzheimer’s disease (AD), Parkinson’s disease (PD), and Huntington’s disease (HD). To date, there is evidence that all seven isoforms contribute to cancer development, while SIRT1-3 and 6 contribute to metabolic and neurodegenerative diseases. Modulators of sirtuin activity are being actively explored to understand their biological and molecular mechanisms and potential for the treatment of various diseases. In this review, we begin with a broad discussion of post-translational modifications, protein deacetylation, and the mechanism of action of sirtuins. Next, we discuss the role of sirtuins in cancer, including inhibitors and activators of sirtuin activity as cancer therapies. In addition, we discuss the relationship of sirtuins to metabolic diseases and as possible treatment targets. Finally, we discuss the role of sirtuins in AD, PD, and HD, and sirtuin modulators for treating neurodegenerative diseases.

## 1. Introduction

The central dogma of molecular biology states that DNA is transcribed to mRNA and then translated into proteins. Proteins are components that form the structural framework and drive the functional processes in all living organisms. The human genome contains between ~19,500 and 20,000 protein-encoding genes [1,2]. However, the variety within this proteome can be significantly increased through mechanisms like alternative splicing and post-translational modifications (PTMs, [3]). PTMs are modifications of proteins by chemical addition and covalent attachment of chemical groups to amino acids in proteins or proteolytic cleavage, giving the modified proteins new characteristics, like altered DNA binding, subcellular localization, protein stability, and enzymatic activity [4]. PTMs rapidly alter the activity, structure, and function of proteins. This ability to quickly alter protein properties allows cells to respond effectively to environmental changes and maintain essential cellular physiology. Many PTMs exist, with the most studied being phosphorylation, acetylation, methylation, lipidation, glycosylation, ubiquitination, and sumoylation. There are over 400 different PTMs, and this field is constantly growing [4,5,6]. These modifications increase the complexity and expand the proteome, which enables the precise regulation and diversification of functions [7]. N^ε^-lysine acetylation was first discovered as a modification of histone proteins [8]. Lysine acetylation directly controls transcription by impacting chromatin modifications and the recruitment of transcription factors [5]. It is known that N^ε^-lysine acetylation extends beyond histones and is a common PTM that affects numerous biological pathways [9]. Lysine residues can be acetylated via enzymatic means by lysine acetyltransferases (KATs) or non-enzymatically using acetyl-CoA as a donor molecule in mitochondria, in a spontaneous manner [10]. No matter which mechanism of acetylation occurs, this process is reversible by the action of the lysine deacetylases (KDACs), traditionally and commonly still referred to as histone deacetylases (HDACs, [11]).

## 2. Protein Deacetylation in Human Cells

**Figure 1:**
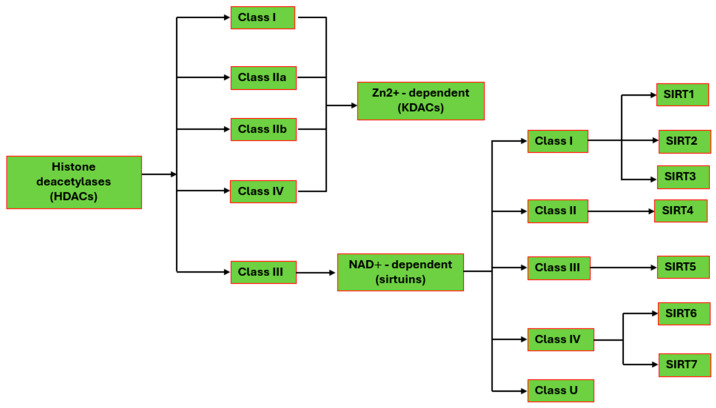
Classification of HDACs based on sequence homology to yeast deacetylases. The HDACs are grouped into four classes: classes I, II, III, and IV. Classes I, II, and IV are comprised of the Zn^2+^ dependent HDACs, and class III is NAD^+^ dependent sirtuins. They are further divided into Classes I, II, III, IV, and U. The 7 human sirtuins fall into classes I–IV. Class U sirtuins are found exclusively in Gram-positive bacteria and contain the conserved catalytic core of ~250–275 amino acids. However, they are phylogenetically distinct from eukaryotic classes I–IV. Unlike human sirtuins, Class U members often lack the regulatory N or C terminal extensions that mediate substrate specificity [14,15].

The human genome encodes a total of 11 HDACs, which are divided into two families, the Zn^2+^-dependent histone deacetylases and the NAD^+^-dependent sirtuins (Figure 1, [12]). Efficient and precise deacetylation is critical for the proper functioning of various biological processes, including transcriptional silencing, DNA recombination and repair, apoptosis, and aging [13].

### 2.1. Zn^2+^-Dependent HDACs

In humans, 11 HDACs have been identified and are grouped into classes based on their sequence homology to yeast deacetylases. Class I enzymes have sequence similarity to yeast Rpd3 and include HDAC1, 2, 3, and 8; class II enzymes have similarity to Hda1, and include HDAC4, 5, 6, 7, 9, and 10. HDAC11 belongs to Class IV proteins and has sequence similarities to class I and class II enzymes (Figure 1, [16]). Class I HDACs mainly target histones and Classes II and IV HDACs target non-histone proteins, including p300, p53, p73, and Ku70 [17,18]. All HDACs share structural similarities and utilize a catalytic Zn^2+^ ion to deacetylate proteins. The majority of class I HDACs are components of multiprotein nuclear complexes that are essential for transcriptional repression and epigenetic landscaping [19], while class II HDACs act as signal transducers that send signals from the cytoplasm to the nucleus. Class IV HDACs are not well-understood, even though their evolutionary conservation suggests that they play important roles in metabolism, immune regulation, and even cell fate and death [20,21]. Human HDACs have a conserved catalytic domain and exhibit substrate specificity in their interactions with target proteins [22].

### 2.2. The Human Sirtuins

**Figure 2:**
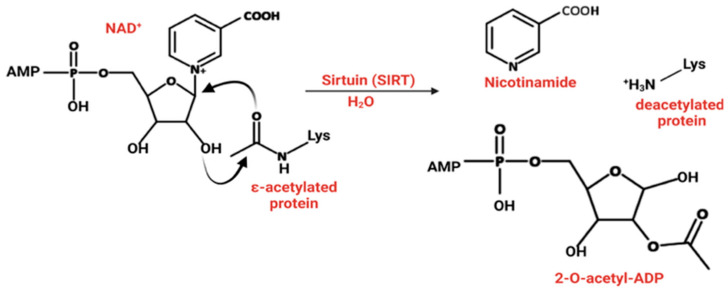
The mechanism of action of NAD^+^-dependent sirtuins. Created in BioRender. Carabetta, V. (2025) https://BioRender.com/ceukoxr.

Sir2p in *Saccharomyces cerevisiae* was initially identified as a silencing factor that was an essential component of heterochromatin and was responsible for repressing transcription at silent mating-type loci, telomeric regions, and the ribosomal DNA array. Beyond its role in transcriptional silencing, Sir2 suppresses recombination within ribosomal DNA repeats, thereby maintaining genomic stability. We now know that Sir2 functions as NAD^+^-dependent sirtuin [23,24]. Sirtuins catalyze the removal of acetyl groups from the side chain of lysine residues, utilizing NAD^+^ as a cofactor, connecting their activity directly to the intracellular concentration of NAD^+^. In this reaction, the acetyl group is transferred to the ADP-ribose moiety of NAD^+^ to form O-acetyl-ADP-ribose, releasing nicotinamide (Figure 2, [25]). Sirtuin substrate specificity is closely tied to their structural features. The highly conserved catalytic core binds NAD^+^ and the acetyl-lysine substrate, while variations in the N and C-terminal extensions determine the isoform-specific substrate recognition and cellular localization. For example, SIRT6 concurrently binds to DNA and the nucleosome acidic patch, which orients the enzyme to selectively access and deacetylate H3 histones [26]. This links their activity directly to metabolic and redox states, thus providing a unique regulatory axis. Unlike HDACs, sirtuins require NAD^+^ as a cofactor for deacetylation. This reaction is feedback inhibited by nicotinamide (NAM) in a non-competitive manner, probably through the base-exchange pathway. NAM binds to the enzyme-NAD^+^ intermediate, effectively reversing the reaction or preventing turnover. NAM inhibits SIRTs 1–3 and SIRT5 with an IC_50_ value ranging from 50 to 184 µM [27].

Sirtuins from eukaryotes, archaea, and bacteria are grouped into five classes based on their sequence homology, structural similarities, and their enzymatic activity with different substrates. There are seven isoforms in humans (Figure 1 and Table 1 [28]). SIRT1-3 belongs to class I, SIRT4 is in class II, SIRT5 is in class III, and SIRT6 and 7 belong to class IV [29,30]. Sirtuins exhibit diverse enzymatic activities across the classes. SIRT1–3 are strong deacetylases, while SIRT4 has weak deacetylase activity and functions as an ADP-ribosyltransferase, lipoamidase, and deacylase [31,32]. SIRT5 has low deacetylase activity, but is a potent desuccinylase, demalonylase, and deglutarylase. SIRT6 and SIRT7 have moderate deacetylase activity, with SIRT6 also acting as an ADP-ribosyltransferase [33,34,35]. These enzymes are found in distinct subcellular locations, with SIRT1, 6, and 7, predominantly found in the nucleus, while SIRT3–5 are primarily located in the mitochondria. SIRT2 is only found in the cytoplasm. Under circumstances of cellular stress caused by environmental conditions, SIRT3 can move from mitochondria to the nucleus [36,37].

**Figure 3:**
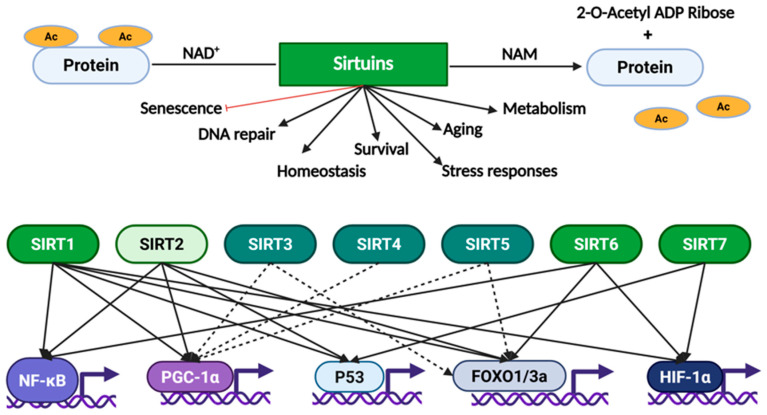
Sirtuins regulate various cellular activities by deacetylating histone and non-histone proteins. Deacetylation requires NAD^+^ as a cofactor and results in the production of nicotinamide (NAM) and 2-O-acetyl ADP ribose. Sirtuins control cellular aging, survival, DNA repair mechanisms, stress response, homeostasis, and metabolism. The seven isoforms are color coded by nuclear (green), cytoplasmic (light green), or mitochondrial localization (teal). Solid arrows represent direct regulation of five important transcription factors (NF-κB, PGC-1α, p53, FOXO1/3a, or HIF-1α, [40]). Dashed arrows represent indirect regulation of mitochondrial sirtuins via control of metabolism or ROS levels. Created in BioRender. Carabetta, V. (2025) https://BioRender.com/nb2ds16.

Sirtuins regulate histones, metabolic enzymes, and transcription factors such as p53, Forkhead box O (FOXO), Peroxisome proliferator-activated receptor gamma coactivator 1α (PGC-1α), and Nuclear factor-kappa B (NF-kB) to regulate cellular functions including DNA repair, metabolism, aging, survival, homeostasis, and stress responses (Figure 3). For instance, SIRT1 deacetylates FOXO, thereby activating it and influencing stress resistance and cell survival [38,39].

## 3. Sirtuins and Cancer

Targeting sirtuins may have the potential to treat various cancers, including breast cancer, lung cancer, colorectal cancer, gastric cancer, prostate cancer, liver cancer, and glioma. In the next section, we explore the known roles of sirtuins in cancer development and discuss currently available or in development sirtuin modulators as potential cancer therapies.

### 3.1. SIRT1, a Contextual Oncogene

Human sirtuins regulate metastasis and the growth of tumors. SIRT1 plays a role in the development, propagation, and advancement of multiple malignant tumors, such as lung, breast, prostate, leukemia, colon, melanoma, ovarian, and gastric cancers [41]. In lung cancer, SIRT1 stimulates cell growth and has a pro-tumorigenic effect. SIRT1 may function also function as a tumor suppressor depending on the signaling pathways it targets, the significance of which may vary depending on the type of cancer [42]. For example, acetylation of β-catenin, a protein involved in Wnt signaling, at lysine 345 is removed by SIRT1, which inhibits its ability to trigger transcription and promote proliferation. SIRT1 is often mutated/deleted in intestinal cancers, and restoration of SIRT1 promotes the cytoplasmic retention of β-catenin. The ability of SIRT1 to promote cytoplasmic retention of β-catenin has therapeutic implications, as it suggests that SIRT1 activators may be useful in treating intestinal cancers. However, overexpression of SIRT1 can also promote tumorigenesis. SIRT1 deacetylates p53 at lysine 382, which blocks p53-dependent pathways and results in uncontrolled cell growth and the inhibition of apoptosis [43]. FOXO transcription factors function as critical tumor suppressors by regulating genes involved in cell cycle arrest, apoptosis, and DNA repair. SIRT1 modulates FOXO activity through deacetylation, thereby suppressing FOXO1-induced apoptosis in prostate cancer cells and promoting FOXO3a ubiquitination and proteasomal degradation [27]. Tumors overexpressing SIRT1 are frequently resistant to chemotherapy, which increases morbidity and mortality for patients [44].

### 3.2. SIRT2, a Second Context-Dependent Regulator in Cancer

SIRT2 not only directly influences the cell cycle, but by modulating the tumor microenvironment, it impacts tumor cell invasion and metastasis. SIRT2 stimulates proliferation by enhancing cell energy metabolism, mediating immune evasion, and altering the extracellular pH. SIRT2 can also be inhibitory towards tumor cells by altering the microenvironment and preventing fibroblast activity, angiogenesis, and other processes [45]. The process of angiogenesis is essential for providing oxygen and nutrients to the growing mass of cells. The enzyme ATP-citrate lyase is required for both cell proliferation and membrane expansion. It is deacetylated by SIRT2, which decreases its stability and inhibits proliferation [46]. The mechanism above has been observed in various types of cancer, including non-small cell lung cancer and esophageal squamous cell carcinoma [47]. SIRT2 promotes liver cancer development in different ways. The protein phosphoenolpyruvate carboxykinase 1 is deacetylated by SIRT2, which increases the activity of the protein. This regulates gluconeogenesis, controlling glucose production in the body when glucose levels are low. Cells are triggered to utilize glucose as an energy source, facilitating cell growth and the progression of tumor metastasis [48]. SIRT2 also decreases the strength of cell adhesion in tissues by blocking the E-cadherin pathway [45]. SIRT2 overexpression in liver cancer cells leads to overexpression of the β-catenin signaling pathway, which enhances the epithelial–mesenchymal transition (EMT). When EMT-related genes are expressed, intercellular adhesion is decreased, which enhances aberrant cancer cell migration and proliferation. Additionally, SIRT2 contributes to tumorigenesis and prognosis of breast cancer, and depending on the tumor grade, it can either promote or suppress growth. For instance, peroxiredoxin-1 is an antioxidant involved in reducing reactive oxygen species (ROS) in cells, helping to maintain cellular redox balance. SIRT2 deacetylates and reduces peroxidase activity, thereby leading to increased levels of ROS within the cell. The increased levels of ROS make certain breast cancer cell lines more susceptible to oxidative stress and DNA damage [49,50]. Pyruvate kinase M2 is a pivotal regulator of aerobic glycolysis and is often upregulated or differentially regulated in cancer cells. It exists in two forms: an active tetramer and a less-active dimer. The dimeric form promotes the diversion of glycolytic intermediates into biosynthetic pathways and supports lactate production, aiding cancer cell proliferation [51]. SIRT2 deacetylates pyruvate kinase M2 at lysine 305, promoting its tetrameric form, which enhances enzymatic activity, increases glycolytic flux, and supports ATP production. SIRT2 knockout reduces pyruvate kinase M2 activity and favors its dimeric form, which promotes lactate accumulation and the diversion of glycolytic intermediates into biosynthetic pathways. This leads to metabolic reprogramming and facilitates the Warburg effect, a hallmark of cancer metabolism, which is characterized by increased glucose uptake and lactate production [52].

### 3.3. The Roles of SIRT3 in Cancer Are Also Context-Dependent

SIRT3 exhibits context-dependent roles in cancer, similar to SIRT1 and SIRT2 [53]. In tumors that rely heavily on mitochondrial oxidative phosphorylation (OXPHOS), SIRT3 can function as an oncogene. Overexpression of SIRT3 enhances mitochondrial respiration and reduces ROS production, thereby promoting tumor growth [54]. Conversely, for tumors that depend on increased glycolytic activity, SIRT3 functions as a tumor suppressor. In the absence of SIRT3, ROS levels become elevated, which in turn triggers the activation of hypoxia-inducible factor 1α (HIF1α) and increases the expression of dependent genes, such as those related to glycolysis and angiogenesis [55]. Experimental evidence supports this dual role. In cultured colon cell lines, a deficiency in SIRT3 increases apoptosis, while reducing cell proliferation, invasion, and migration [56], whereas breast cancer cells with upregulated SIRT3 exhibit decreased glycolysis and proliferation, providing a metabolic route for tumor suppression [57].

SIRT3 exerts these effects by regulating mitochondrial dynamics, which encompass mitochondrial biogenesis, fusion, fission, and selective degradation via mitophagy. Under normal metabolic conditions, SIRT3 associates with ATP synthase to sustain OXPHOS through the electron transport chain. However, loss of mitochondrial membrane potential or pH imbalance triggers SIRT3 dissociation, enabling quality control mechanisms that restore mitochondrial integrity. SIRT3 drives mitochondrial biogenesis via deacetylation of optic atrophy 1 (OPA1), which orchestrates mitochondrial fusion, maintains cristae morphology, and protects cells from apoptotic stress. Additionally, SIRT3 also influences mitochondrial fission by modulating the expression and activity of dynamin-related protein 1 and mitochondrial fission 1 protein through FOXO3a deacetylation and AMPK-PGC-1α signaling. Depending on the cellular context, this regulation can either promote or suppress fission, suggesting that SIRT3 fine-tunes mitochondrial morphology, bioenergetic efficiency, and ultimately tumor cell fate [58,59].

### 3.4. The Roles of SIRT4 in Cancer

SIRT4 may have both oncogenic and tumor suppressor effects in cancers, although the mechanisms of action remain unclear [60]. The pattern of SIRT4 expression in breast cancer is controversial. Although some studies indicate an upregulation of SIRT4 expression, others report downregulation, which may be due to the variations in the breast cancer cell types or the methodologies used [61]. This discrepancy precludes the possibility of targeting SIRT4 in the treatment of breast cancer. However, it may be more promising as a drug target for prostate cancer. SIRT4 inhibits the uptake of glutamine, a metabolite essential for the proliferation of tumor cells, which in turn limits the growth of prostate cancer cell lines [61]. SIRT4 stimulates mitochondrial-mediated death in prostate cancer cell lines by deacetylating adenine nucleotide translocase-2, which is highly expressed in the mitochondrial inner membrane in cancers, and triggering its degradation [62]. SIRT4 also exhibits tumor suppression effects in other cancers, such as thyroid cancer, colorectal cancer, and B-cell lymphoma [63].

### 3.5. SIRT5 Plays a Dual Role in Cancer

**Figure 4:**
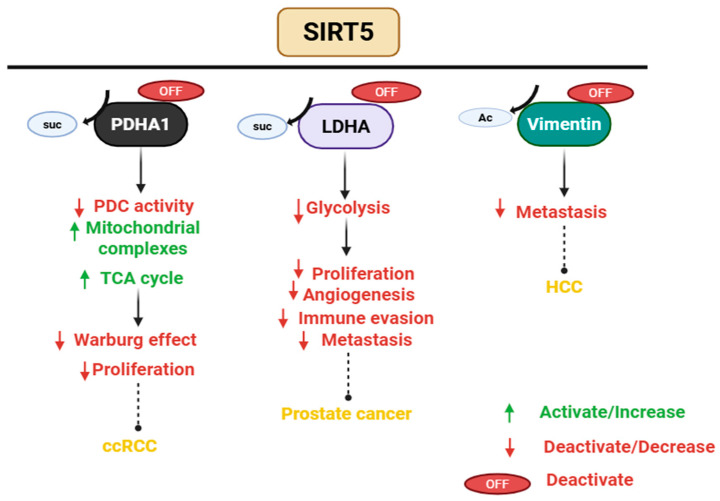
SIRT5 acts as a tumor suppressor in cancer cells. SIRT5 is a desuccinylase and deacetylase, which inactivates its target proteins, including pyruvate dehydrogenase complex E1α subunit (PDHA1), lactate dehydrogenase A (LDHA), and Vimentin. This prevents immune evasion, cell proliferation, metastasis, and angiogenesis. PDC, pyruvate dehydrogenase complex; clear cell renal cell carcinoma, ccRCC, hepatocellular carcinoma, HCC. Created in BioRender. Carabetta, V. (2025) https://BioRender.com/848xp5h.

SIRT5 regulates multiple mitochondrial metabolic activities, including fatty acid oxidation, glycolysis, regulation of amino acid breakdown, and cellular respiration (Figure 4, [64]). SIRT5 serves as a tumor suppressor by inhibiting cancer cell proliferation and metastasis, enhancing resistance to ROS, and inhibiting the Warburg effect [65]. These functions are particularly evident in clear cell renal cell carcinoma, gastric cancer, hepatocellular carcinoma, and prostate cancer. For instance, in clear cell renal cell carcinoma, SIRT5 promotes mitochondrial OXPHOS by desuccinylating enzymes such as pyruvate dehydrogenase complex E1α subunit (PDHA1), which shifts metabolism away from aerobic glycolysis and restrains tumor growth. SIRT5 also targets lactate dehydrogenase (LDHA), a critical enzyme in glycolysis that is frequently upregulated and hyper-succinylated in cancer cells. SIRT5-mediated desuccinylation at lysine 118 reduces its enzymatic activity, thereby attenuating glycolytic flux and lactate production. Functionally, this results in impaired proliferation and metastatic potential of prostate cancer cells [66]. Silencing SIRT5 in gastric cancer cell lines increased the rate of cell proliferation, migration, invasion, and tumor formation in immunodeficient mice [67]. Additionally, in hepatocellular carcinoma cell lines, SIRT5 directly interacts with vimentin and catalyzes its deacetylation at lysine 120, a modification that attenuates vimentin function and subsequently suppresses cell migration. Acetylation at this residue enhances vimentin activity, thereby facilitating EMT and promoting migratory behavior. The acetylation-dependent control of vimentin by SIRT5 underlies its inhibitory role in EMT and motility in cancer cells [68].

**Figure 5:**
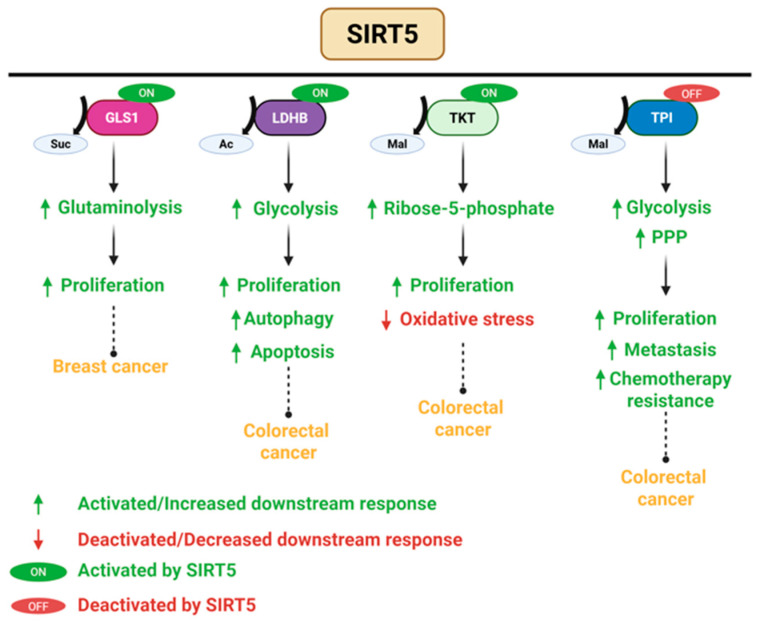
SIRT5 acts as an oncogene in breast cancer and colorectal cancer. SIRT5 enhances the expression of oncogenes or directly interacts with and modifies them, thereby facilitating cancer cell proliferation, survival, chemoresistance, and metastasis. The red circle with OFF refers to inactivation by SIRT5, while the green circle with ON refers to activation. GLS1, glutaminase 1; LDHB, lactate dehydrogenase B; TKT, transketolase; and PPP, pentose phosphate pathway. Created in BioRender. Carabetta, V. (2025) https://BioRender.com/erioztm.

SIRT5 upregulates the expression of several oncogenes, thus enhancing cell survival, resistance to chemotherapy, proliferation, and metastasis (Figure 5, [65,69]). In breast cancer, SIRT5 promotes tumor growth by desuccinylating and stabilizing glutaminase 1, thereby enhancing its enzymatic activity. This modification facilitates glutaminolysis, increasing the availability of glutamate and downstream α-ketoglutarate, fueling the tricarboxylic acid (TCA) cycle and supporting the biosynthetic demands of rapidly proliferating cancer cells [70,71]. Additionally, SIRT5 acts as a deacetylase, desuccinylase, and a demalonylase to activate target proteins such as lactate dehydrogenase B, and transketolase, promoting cell proliferation, oxidative stress, and cell survival in colorectal cancer [65,72]. For instance, SIRT5 expression promotes colorectal cancer cell survival by stimulating autophagy through the deacetylation of lactate dehydrogenase B, which facilitates lysosomal acidification [73]. Additionally, SIRT5 regulates glutamine metabolism. Silencing of SIRT5 disrupts the conversion of glutamate to α-ketoglutarate, thereby limiting the flow of glutamine-derived carbon into the TCA cycle. This metabolic disruption impairs anabolic biosynthesis, ultimately suppressing cancer growth and proliferation [74]. Transketolase is an enzyme involved in the non-oxidative arm of the pentose phosphate pathway. SIRT5 activates it by lysine 281 demalonylation, producing ribose-5-phosphate for nucleotide synthesis and preserving NADPH levels for antioxidant defense and reductive biosynthesis. This activation promotes the progression of colorectal cancer in vitro and in vivo. In mouse xenograft models, colorectal cancer cells with enhanced SIRT5-transketolase signaling exhibited accelerated tumor growth. Overexpressing SIRT5 leads to a poor prognosis for colorectal cancer patients [75]. In addition, SIRT5 catalyzes the demalonylation of triose phosphate isomerase at lysine 56 to suppress its enzymatic activity. As a result, glucose metabolism is shifted toward the oxidative pentose phosphate pathway, enhancing NADPH production and enabling cancer stem cells to resist chemotherapy-induced ROS [65]. Functional suppression of SIRT5 disrupts tumorigenic processes by inhibiting cell proliferation and apoptosis, highlighting it as a candidate for targeted therapy.

### 3.6. The Context Dependent Role of SIRT6 in Cancer

SIRT6 exhibits multiple enzymatic activities, including low levels of ADP-ribosylation and deacetylation. More recently, SIRT6 was shown to possess deacylase activity, enabling the removal of long-chain fatty acyl groups such as myristoyl and palmitoyl moieties from lysine residues, thereby influencing protein function and subcellular localization [76]. Although SIRT6 is primarily located in the nucleus, where it regulates chromatin structure and transcription, it is also found in the endoplasmic reticulum. Functionally, SIRT6 maintains cellular homeostasis through the regulation of metabolic pathways, DNA damage repair, inflammation, and telomere maintenance. Its role in cancer is complex depending on the cellular environment and cancer type [77]. As a tumor suppressor, SIRT6 represses tumor growth in several cancers including breast and pancreatic cancer, primarily by inhibiting aerobic glycolysis [43]. By inhibiting the enzymatic activity of specific glycolytic genes, SIRT6 disrupts the energy metabolism required for rapid tumor cell proliferation [78]. Conversely, in lung cancer, prostate cancer, melanoma, and non-melanoma skin cancers, SIRT6 levels are increased, which promotes tumor progression. In this case, SIRT6 may support oncogenic growth by enhancing DNA repair capacity, maintaining genome stability, and supporting anabolic metabolism [79].

### 3.7. The Role of SIRT7 in Cancer

SIRT7 regulates several pathways that include ribosome biogenesis, transcription, aging, genomic stability, metabolism, and carcinogenesis [80]. SIRT7 is overexpressed in multiple cancer types, including bladder, leukemia, prostate, and breast cancer [81,82]. In osteosarcoma cells, knocking down SIRT7 decreased proliferation, migration, invasion, and growth, whereas overexpression had the opposite effects. From a mechanistic standpoint, SIRT7 deacetylates histone H3 at lysine 18, which represses CDC4 transcription. CDC4 functions as the substrate recognition subunit within the SCF E3 ubiquitin ligase complex, regulating a broad set of proteins essential for cell division, growth, and differentiation. Recognized as a tumor suppressor, CDC4 is frequently mutated or deleted in human cancers [41,82,83]. SIRT7 accelerates the proliferation, migration, and invasion of osteosarcoma cells through the inhibition of CDC4, suggesting that SIRT7 could serve as a potential therapeutic target. Additionally, SIRT7 directly deacetylates p53 to prevent apoptosis in response to stress and indirectly regulates its stability [84]. p53 is important for cellular responses to DNA damage and other stressors, which causes cell cycle arrest, aids in DNA repair, and activates apoptosis to destroy cells that have accumulated large levels of damaged DNA [85]. SIRT7 regulates p53 stability during stress responses triggered by ultraviolet radiation, by inhibiting ubiquitination [86,87]. In the absence of SIRT7, the stabilization of p53 in response to cellular stress is compromised, thereby impairing the activation of stress-responsive pathways and diminishing the cellular ability to cope with stress. Stabilization of p53 is a mechanism exploited by numerous anti-tumor drugs. However, in tumors harboring oncogenic mutations in the p53 gene, this stabilization may be detrimental. SIRT7 inhibition may promote degradation of mutant forms of p53, while its activation could stabilize wild-type p53, especially under stress-inducing conditions [86].

### 3.8. Small-Molecule Modulators of Sirtuins as Cancer Therapy

Therapies targeting sirtuins in cancer exploit their multifaceted roles in tumor development, using inhibitors or activators to modulate their activity and potentially boost the success of current cancer treatments. Here, we discuss the mechanisms of sirtuin inhibitors and activators and explore their anti-tumor effects and potential as effective treatments.

#### 3.8.1. Sirtuin Inhibitors as Cancer Therapy

**Figure 6:**
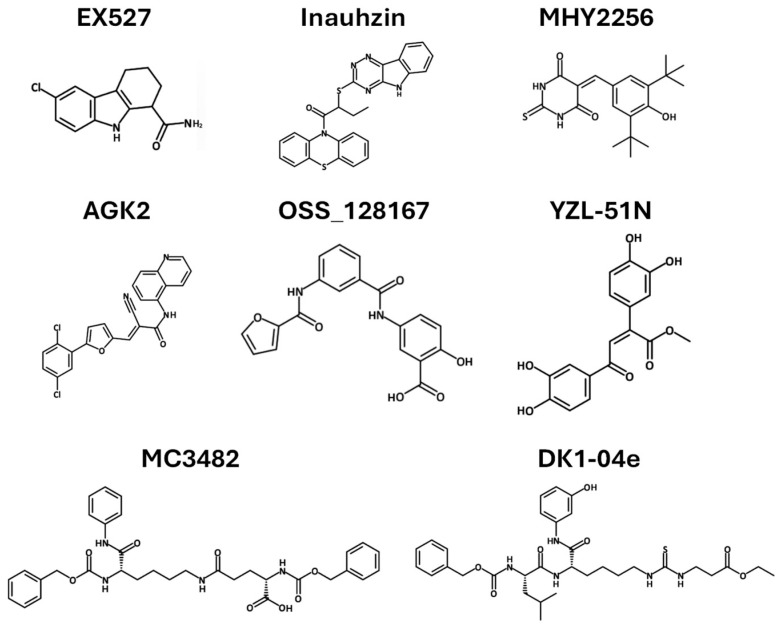
Chemical structure of sirtuin inhibitors. Structures drawn using Mavin Chemical Sketch.

Inhibitors of the Sirtuin–p53 pathway have been explored in preclinical studies, revealing several promising anticancer drug leads. EX527 is a cell-permeable, selective SIRT1 inhibitor (IC_50_ = 98 nM) with minimal activity against SIRT2/3 and no effect on other HDACs (Figure 6 and Table 2). It exerts anti-tumor effects in glioma cells, resulting in the accumulation of acetylated p53 and subsequent transcriptional induction of p21. The upregulation of p21 facilitates cell cycle arrest, whereas prolonged activation of p53 signaling drives apoptosis in glioma cells [88,89]. This suggests that EX527 could serve as a potential agent for glioma therapy. Inauhzin also inhibits SIRT1and has a p53-dependent anti-tumor activity in xenograft lung and colon cancer models, with high selectivity for cancer cells (Figure 6 and Table 2). Mechanistically, Inauhzin non-competitively inhibits NAD^+^ binding to SIRT1, which suppresses its activity, and prevents MDM2-mediated p53 ubiquitination by increasing p53 acetylation. Preclinical studies of its analog, Inauhzin-C, established maximum tolerated doses, without significant biochemical or pathological changes. However, dose-dependent reductions in alkaline phosphatase and bilirubin suggest potential hepatotoxicity, warranting further investigation [90,91].

**Table 2:** Sirtuin inhibitors as cancer therapies.

Compound	Target	Cancer Type	Mechanism of Action	REF
EX527	SIRT1	Glioma	Upregulates p53, increases acetylated p53 and p21, induces apoptosis	[88]
Inauhzin	SIRT 1	Lung Colon	Inhibits the binding of NAD^+^ to SIRT1, inhibits SIRT1 activity and blocks MDM2-mediated ubiquitination, triggers p53-dependent apoptosis	[90,91]
MHY2256	SIRT1-3	Breast	Reduces SIRT1-3 levels, increases p53 acetylation, induces apoptosis	[92,93]
AGK2	SIRT2	Breast	Suppresses cell proliferation and viability, and induces cell cycle arrest and apoptosis	[94]
DK1-04e	SIRT5	Breast	Reduces tumor burden and total tumor weight	[95]
MC3482	SIRT5	Breast	Increases intracellular ammonia and promotes ammonia-induced autophagy	[74,96]
OSS_128167	SIRT6	Large B-cell lymphoma	Decreases cell proliferation, induces cell apoptosis, and blocks cell cycle	[97]
YZL-51N	SIRT7	Colon	Suppresses DNA repair, increases chromatin instability	[98]

MHY2256 is a novel SIRT inhibitor that exhibits anti-cancer effects by promoting p53 acetylation (Figure 6 and Table 2, [93]). MHY2256 has been shown to reduce breast cancer cell viability in preclinical studies by downregulating the expression of SIRT1, SIRT2, and SIRT3. This reduction in SIRT levels leads to increased levels of acetylated p53. Increased p53 acetylation enhances its pro-apoptotic activity, ultimately promoting cell death in the cancerous cells. MHY2256 demonstrates significant cytotoxicity in cancer cells [92], but is lacking safety evaluations using animal models.

AGK2 is a selective and potent SIRT2 inhibitor that targets the active site, inhibiting its deacetylase activity and modulating downstream protein interactions (Figure 6 and Table 2). AGK2 exhibits therapeutic potential across a range of in vitro and in vivo models. In an in vitro assay, AGK2 inhibited cell proliferation and induced G1 phase arrest by downregulating the expression of CDK4 and CDK6. Furthermore, AGK2 suppresses the expression of p53, suggesting a broader impact on cell cycle regulation and stress response pathways [99]. Paclitaxel (PAX) is a widely used chemotherapeutic agent for the treatment of breast cancer. Despite its widespread use for breast cancer, PAX is often considered last resort due to serious side effects such as neurotoxicity, cardiotoxicity, and hematological toxicity. For breast cancer cell lines, treatment with either PAX or AGK2 alone suppressed cell proliferation and viability and induced both cell cycle arrest and apoptosis. In addition, AGK2 synergistically increased the anticancer efficacy of PAX in most breast cancer cell lines. This suggests that AGK2 and PAX may be more effective as combinatorial therapy. However, the underlying molecular mechanisms of synergy at the cellular level are not understood, and potential adverse effects need to be thoroughly assessed [94].

DK1-–04e is a selective, cell-permeable, and potent small-molecule SIRT5 inhibitor that effectively inhibits the growth of breast cancer cells in vitro and significantly suppresses mammary tumor development in vivo (Figure 6 and Table 2). Pharmacological inhibition of SIRT5 was evaluated in vivo using a murine breast cancer model. Following the initial detection of palpable mammary tumors, mice were treated with intraperitoneal injections of either vehicle or DK1-04e. DK1-04e treatment significantly reduced tumor burden and total weight compared to vehicle-treated controls [95]. Another selective inhibitor of SIRT5, MC3482, led to increased levels of intracellular ammonia, inducing autophagy and mitophagy through ammonia signaling in cell lines [74,96]. The effects of MC3482 have not been explored with animal models. Since autophagy can both promote and suppress tumor development depending on context, the effects of MC3482 on breast and other cancers should be investigated.

To date, only a few SIRT6 inhibitors have been developed, and those available demonstrate limited potency (Figure 6 and Table 2). Their anti-tumor effects are relatively weak, with the underlying mechanisms largely unexplored [100]. The novel small-molecule compound OSS_128167 selectively inhibits SIRT6 and promotes chemosensitization in multiple myeloma cells. OSS_128167 also decreased cell proliferation, induced apoptosis, and blocked the cell cycle in diffuse large B-cell lymphoma cell lines. In an in vivo xenograft model, mice were treated with OSS_128167 or a vehicle control via intraperitoneal injection. A substantial reduction in tumor growth was observed in mice treated with OSS_128167, consistent with the in vitro results [97]. Further investigations to fully understand the mechanism of action and spectrum of activity are necessary.

YZL-51N is a selective inhibitor of SIRT7, exhibiting an IC_50_ of 12.7 µM. It interferes with SIRT7 activity by competing for the NAD^+^ binding site, which ultimately impairs DNA damage repair processes and reduces the survival of cancer cells (Figure 6, Table 2). YZL-51N disrupts DNA repair mechanisms and elevates chromatin instability. In colorectal cancer cells, YZL-51N inhibited DNA repair mechanisms and showed a synergistic therapeutic effect in combination with etoposide. Etoposide is a commonly used chemotherapy that binds to the DNA-topoisomerase II complex, inhibiting the resealing of cleaved DNA strands, leading to prolonged DNA damage. High concentrations of YZL-51N reduced cell growth and colony formation in multiple colorectal cancer cell lines. Moreover, in a xenograft mouse model, subcutaneous administration of YZL-51N and 3 Gy ionizing radiation, alone or in combination, led to a decrease in tumor volume relative to the control group [98]. Future studies should focus on understanding the underlying mechanisms of action for YZL-51N in cancer cells, as well as a comprehensive evaluation of its toxicity profile.

#### 3.8.2. Sirtuin Activators as Cancer Therapy

**Figure 7:**
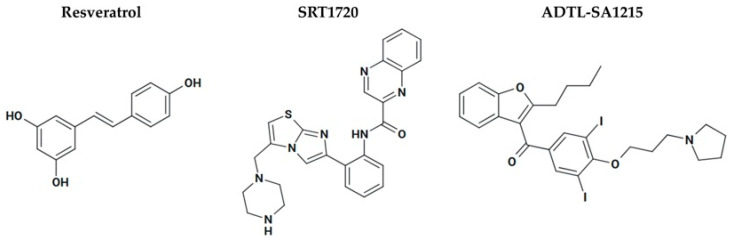
Chemical structure of sirtuin activators. Structures drawn using Mavin Chemical Sketch.

Sirtuin activators are emerging as promising anticancer agents due to their involvement in the regulation of cell survival, metabolism, DNA repair, and aging. Resveratrol, a polyphenol found naturally in grapes, red wine, peanuts, and berries, is one of the most intensively studied SIRT1 activators (Figure 7 and Table 3). Its anti-cancer effects have been demonstrated in both experimental cell cultures and animal models. Resveratrol inhibited the growth of human skin cancer cell lines by decreasing the expression levels of cyclins D1, D2, and E, as well as reducing the activity and/or expression of CDK2, CDK4, and CDK6. It also increased the expression of p21 [101,102]. Similar antiproliferative effects were observed in breast cancer and prostate cancer cell lines, where resveratrol regulated CDK4 and cyclin D1, potentially via activation of the p21 and p53 pathways [102,103]. In xenograft models, resveratrol suppressed estrogen receptor (ER)-β–positive, ER-α–negative tumor growth, increased apoptosis, and reduced angiogenesis in nude mice [104]. However, it showed no effect on ER-α–negative tumor growth or metastasis at low intraperitoneal doses, suggesting dose insufficiency. At higher oral doses, tumor growth and lung metastasis were inhibited, likely through the downregulation of matrix metalloproteinase-9 [102,105], which is highly expressed in invasive tumors and is thought to contribute to cancer invasion by enzymatically breaking down the extracellular matrix. In androgen receptor-negative (AR-) human prostate cancer cell xenografts implanted in the flank regions of mice, oral administration of resveratrol led to reduced tumor volume, decreased tumor cell proliferation, and promoted apoptosis. Similarly, intraperitoneal administration of resveratrol in an orthotopic prostate cancer model resulted in inhibited tumor growth, slowed progression, reduced local invasion, and lowered spontaneous metastasis [106]. Resveratrol has been studied in human clinical trials to assess the pharmacokinetics, toxicity, and metabolism in both healthy and cancer patients. Resveratrol is extensively metabolized and excreted in the urine, leading to poor bioavailability. As a result, higher doses were administered and found to be generally safe. Adverse gastrointestinal effects such as nausea, diarrhea, and abdominal pain were observed. A 5% decrease in tumor cell proliferation in colorectal tissue was observed, based upon the expression of the proliferation marker Ki-67 [107,108].

**Table 3:** Summary of sirtuin activators as cancer therapy.

Compound	Models Studied	Mechanism of Action	Outcome	REF
Resveratrol	A431 human skin cancer cells	↓ Cyclin D1, D2, D3, E; ↓ CDK2, CDK4, CDK6; ↑ p21 expression	Inhibited tumor growth	[101,102]
	MCF-7 breast cancer cells DU-145 prostate cancer cells	Regulation of CDK4 and cyclin D1, ↑ p21 and p53 pathways	Antiproliferation	[102,103]
	MDA-MB-231 xenografts (ER-β+, ER-α−, nude mice)	↑ apoptosis, ↓ angiogenesis	Inhibited tumor growth	[104]
	4T1 breast cancer xenografts (ER-α−)	↓ MMP-9	Inhibited tumor growth ↓ lung metastasis	[105]
	PC-3 prostate cancer xenografts (AR-)	↓ tumor volume and proliferation, ↑ apoptosis	↓ tumor volume, ↓ proliferation, ↑ apoptosis	[106]
	DU-145 orthotopic prostate cancer (mice)	↓ tumor volume, invasion, proliferation, metastases	Inhibited tumor growth, ↓invasion, ↓ metastasis	[106]
	Clinical (healthy and cancer patients)	PK, rapid metabolism, poor bioavailability	Safe up to 5 g/day, ↓ Ki-67 in colorectal tissue	[107,108]
SRT1720	Bladder cancer cells	Blocks late-stage autophagy (↓ fusion with lysosomes), deacetylates LAMP2	Inhibited migration/invasion, ↑ apoptosis	[109]
	Bladder cancer organoid cultures and mouse models	SIRT1 deacetylates HIF-1α ↓ hypoxia signaling	Suppressed tumor growth	[110]
	Multiple myeloma cell line	ATM-dependent apoptosis; Caspase activation; DNA damage, ER stress, ↑ ROS; inhibition of NF-κB and VEGF signaling	Selective toxicity (IC_50_: 3–7 µM); ↓ tumor growth; synergistic with bortezomib/dexamethasone	[111]
ADTL-SA1215	MDA-MB-231 human breast cancer cells; xenografts	↑ SIRT3-mediated autophagy and mitophagy; suppression of proliferation and migration	Inhibited tumor growth and migration	[112,113]

↑ Increased level or activity; ↓ Decreased level or activity. CDK, cyclin-dependent kinase; AR-, androgen receptor-negative, ER, estrogen receptor.

SRT1720 is a synthetic small molecule SIRT1 activator that has anti-cancer activity in preclinical models (Figure 7 and Table 3). SRT1720 treatment inhibited the migration and invasion of bladder cancer cells and promoted apoptotic cell death. Mechanistically, SRT1720 promotes the accumulation of autophagosomes by initiating early-stage autophagy but impairing late-stage autophagy by blocking their fusion with lysosomes. SRT1720 enhances the expression of autophagy-related proteins and modifies their acetylation status, thereby impairing autophagic flux. Lysosomal-associated membrane protein 2 (LAMP2) may be involved in this process, as SRT1720 treatment led to significant deacetylation, potentially affecting its functional role in autophagy. LAMP2 regulates autophagy, lysosomal stability, and cellular homeostasis [109]. SIRT1 mutation promotes cancer proliferation and diminishes their sensitivity to SRT1720, supporting SIRT1 as a direct and relevant target of SRT1720 in bladder cancer. The anti-tumor activity of SRT1720 is mimicked by genetic or pharmacological inhibitions of HIF [110]. SRT1720 induces toxicity in multiple myeloma cells through an ATM-dependent pathway in in vitro and in vivo models. SRT1720 reduces cancer cell viability after 24 h of treatment, with minimal toxicity to normal peripheral blood mononuclear cells, highlighting its selective cytotoxicity and potential for targeted therapy. Mechanistically, SRT1720 initiates apoptosis in myeloma cells through caspase activation, DNA damage, and inhibition of NF-κB signaling. In addition, treatment with SRT1720 significantly inhibited multiple myeloma tumor growth compared to vehicle controls in a human plasmacytoma xenograft mouse model. Furthermore, combining SRT1720 with either bortezomib or dexamethasone produced synergistic anti-myeloma effects [111]. Despite SRT1720 showing anti-cancer effects in vitro and in vivo, there are no human clinical trials reported to date.

Activation of SIRT3 may be a viable therapeutic option for specific highly malignant tumors, particularly for those that are resistant to available chemotherapies. Sorafenib is an oral anti-cancer drug that is a kinase inhibitor, and is used to treat liver, thyroid, and renal cancer. Resistance to Sorafenib develops rapidly, and its efficacy in treating liver cancer is limited [114]. One effect of Sorafenib is the downregulation of SIRT3 expression, which is likely to decrease drug sensitivity. Upregulation of SIRT3 has been shown to enhance mitochondrial function, reduce ROS levels, and restore sensitivity to sorafenib in hepatocellular carcinoma cell lines. These findings suggest that SIRT3 modulates the metabolic response to sorafenib, and that therapeutic strategies aimed at increasing SIRT3 expression may enhance treatment efficacy [115]. Through a structure-guided design and high-throughput screen, ADTL-SA1215 was identified as a selective SIRT3 activator (Figure 7 and Table 3). The small molecule was found to suppress the proliferation and migration of breast cancer cells by modulating SIRT3-mediated autophagy and mitophagy pathways, both in vitro and in vivo [112,113]. These findings suggest that pharmacological activation of SIRT3 could represent a promising therapeutic strategy for triple-negative breast cancer.

#### 3.8.3. Polyphenols That Activate Sirtuins as Possible Anticancer Agents

**Figure 8:**
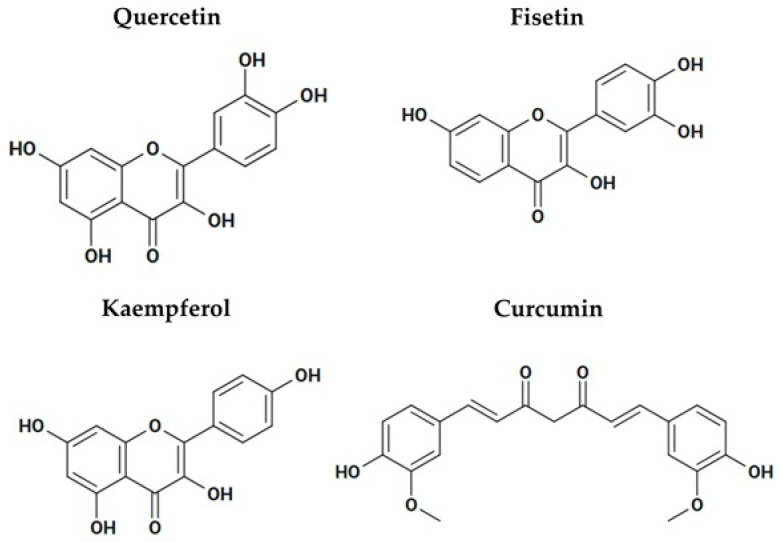
Chemical structures of polyphenols that activate sirtuins and have anticancer activity.

Quercetin is a polyphenol that activates SIRT1 and SIRT6 (Figure 8). Quercetin has anticancer properties in hepatoblastoma cells by inducing SIRT6-mediated deacetylation of H3K9, which ultimately downregulates Wnt/β-catenin signaling. This suppresses proliferation and invasion while enhancing apoptosis, both in vitro and in vivo [116]. Quercetin indirectly activates SIRT1 by elevating NAD^+^ and AMPK activation, which increases autophagy and apoptosis, modulates inflammation and oxidative stress, and enhances genome stability [117]. Quercetin and its derivative KPMF-8 also directly activate SIRT1, which increases its affinity for substrates such as p53. KPMF-8 increased SIRT1 binding affinity for the acetylated p53 peptide by ~8-fold, whereas resveratrol only produced a 1.4-fold increase [118]. Fisetin is another polyphenol that is found in grapes, apples, cucumbers, strawberries, onions, and persimmons (Figure 8). It activates SIRT1 and has anticancer, antioxidant, and anti-inflammatory effects. Fisetin counteracts oxidative stress and modulates immune responses through activation of the AMPK-SIRT1 and Nrf2 signaling pathways [119]. Fisetin suppressed pancreatic cancer progression by targeting the PI3K/AKT/mTOR signaling cascade, thereby inhibiting cell proliferation, migration, and invasion [120]. Kaempferol, a natural flavonoid found widely in fruits, vegetables, and plants exhibit anticancer effects in diverse tumor types (Figure 8). Kaempferol activates SIRT1 and induces apoptosis, suppresses inflammation, and inhibits tumor cell migration. In lung and colorectal cancer, kaempferol suppresses cell proliferation and viability, and in HCC, it exhibits dose and time-dependent inhibition of cell viability [121]. Kaempferol enhances the efficacy of sorafenib chemotherapy when used in combination at subtoxic concentrations [122]. In addition, kaempferol combined with 5-fluorouracil was found to inhibit cell viability more effectively than either agent alone [123]. Curcumin is a polyphenol that activates SIRT1 and AMPK (Figure 8). In head and neck squamous cell carcinoma, curcumin has been shown to suppress cell migration and angiogenesis while promoting apoptosis through the activation of caspase-8 signaling pathways [124]. Curcumin directly binds to SIRT1, reducing its stability and consequently reducing its oncogenic activity. Curcumin induces ubiquitination-dependent proteasomal degradation of SIRT1 [125]. Collectively, the evidence indicates that these polyphenols are promising as anticancer therapies; however, further investigations are required to understand their mechanisms of action, optimize their bioavailability, and evaluate their efficacy in clinical settings.

## 4. Beyond Cancer: The Role of Sirtuins in Metabolic Diseases

Type 2 diabetes mellitus, obesity, insulin resistance, osteoporosis, lipid metabolism disorders, and hyperuricemia are among the metabolic diseases that are prone to developing when the balance between energy intake and expenditure is disrupted, either by hereditary or environmental causes [126]. The overabundance of energy resulting from obesity causes circulating lipids to accumulate in non-adipose tissues and creates pathological abnormalities. In adipose tissue, liver, and muscle, sirtuins modulate insulin resistance and glucose uptake. Here, we discuss the role of sirtuins in diabetes, obesity, and osteoporosis.

### 4.1. The Role of Sirtuins in Diabetes

In diabetes, sirtuins have a protective function by improving insulin sensitivity, reducing inflammation, and regulating glucose metabolism. They modulate blood glucose levels by protecting pancreatic β-cells and regulating insulin secretion. In mice with obesity-related renal disorders, SIRT1 overexpression reduced oxidative stress and cell aging-induced renal pathological damage. The pro-apoptotic gene BAX and NF-κB levels were increased by hyperglycemia and remained high after returning to normoglycemia in diabetic rats and bovine endothelial cells. Sensitivity to hyperglycemic stress was elevated with SIRT1 knockdown [127]. Overexpression or metformin-induced SIRT1 activation inhibits the induction of liver kinase B1/AMP-activated protein kinase, which in turn suppresses the expression of NF-κB and BAX [128]. NF-κB deacetylation mediated by SIRT1 prevents β-cell damage caused by cytokines and nitric oxide in isolated rat islets [129]. Endocrine progenitor differentiation and β-cell recovery are also enhanced by SIRT1 activation [129]. Together, this suggests that SIRT1 activation could be a new diabetes treatment strategy.

The expression of SIRT2 is reduced in insulin-resistant hepatocytes, which is associated with elevated ROS production, activation of the stress-responsive ERK1/2 pathway, and impaired mitochondrial function. Overexpressing SIRT2 enhances insulin sensitivity, reduces ROS levels, and alleviates mitochondrial dysfunction [130]. Moreover, impaired hepatic glucose uptake is a major factor driving postprandial hyperglycemia. In obese diabetic mice, reduced hepatic NAD^+^ lowered SIRT2 activity, which impaired glucose uptake. SIRT2 restored hepatic glucose uptake by deacetylating the glucokinase regulatory protein at lysine 126, releasing glucokinase and improving glucose tolerance and insulin sensitivity [46,131]. SIRT2 haplotypes displayed the opposite effects, indicating their role in modulating susceptibility to type 2 diabetes [132]. This highlights the therapeutic potential of SIRT2 activation in treating diabetes.

SIRT3 regulates mitochondrial metabolism, insulin resistance, and insulin signaling in diabetes mellitus. SIRT3 knockout mice develop insulin resistance due to the hyperacetylation of mitochondrial proteins in skeletal muscles. SIRT3 activity is diminished in diabetic muscle tissue, suggesting a metabolic impairment. Given the critical role of SIRT3 in maintaining mitochondrial function, elucidating its regulatory role in cellular metabolism is essential for understanding the connection between mitochondrial dysfunction and metabolic disorders [133]. SIRT3 also contributes to diabetic osteoporosis, a complication of type 2 diabetes mellitus marked by impaired osteoblast function and disrupted bone microarchitecture. In both the in vitro and in vivo diabetes models, decreased SIRT3 expression was observed, which led to the hyperacetylation of FOXO3 and suppression of the mitophagy pathway. Restoration of SIRT3 activity through overexpression or pharmacological activation reactivated mitophagy and improved osteogenic function [134].

In pancreatic β-cells, SIRT6 activity deacetylates FOXO1, leading to the increased expression of genes that maintain glucose-sensing capacity and support glucose tolerance [135]. SIRT6 deficiency in pancreatic β-cells impairs insulin secretion. Beyond the pancreas, physiological overexpression of SIRT6 enhances insulin sensitivity in skeletal muscle and liver, providing protection against type 2 diabetes mellitus [136]. In mice, SIRT6 deficiency in skeletal muscles impairs glucose homeostasis and insulin sensitivity, reduces energy expenditure, and diminishes exercise performance [137]. Pharmacological inhibition of SIRT6 improves glucose tolerance and increases GLUT1 and GLUT4 expression in muscle, demonstrating that both the activation and inhibition of SIRT6 can have beneficial effects depending on the tissue [138]. These findings underscore SIRT6s dual functions, it generally acts protectively by mitigating metabolic dysfunction, but its effects can vary depending on tissue type and molecular pathways. This duality makes further SIRT6 research necessary before the possibility of it as a therapeutic target can be evaluated. The role of SIRT4, SIRT5, and SIRT7 in diabetes is underexplored, warranting further studies.

### 4.2. The Therapeutic Potential of Sirtuins in Obesity

Obesity is a complex condition marked by excessive body fat accumulation and poses a serious public health concern worldwide. Most studies suggest that SIRT1, SIRT3, and SIRT6 play protective roles in obesity. For instance, SIRT1 plays a key role in lipid metabolism, adipogenesis, and adipokine regulation. SIRT1 targets and deacetylates coactivators of peroxisome proliferator-activated receptor gamma (PPARγ), a key transcriptional regulator of adipogenesis. Through this modification, SIRT1 alters PPARγ activity, thereby suppressing adipocyte differentiation and limiting fat accumulation. The overexpression of SIRT1 or its pharmacological activation has been shown to increase energy expenditure and limit fat accumulation. Studies have shown that mice overexpressing SIRT1 exhibit reduced obesity, with stronger effects in females than males, likely due to the ER-α-mediated induction of SIRT1. Mechanistically, SIRT1 inhibits autophagy and lipid production by deacetylating and activating AKT and STAT3 via the mTOR-ULK1 and p55 pathway [139]. This suggests that activating or overexpressing SIRT1 may reduce obesity.

In obesity, SIRT3 expression is reduced, resulting in the hyperacetylation of mitochondrial proteins, thereby compromising mitochondrial integrity and function. SIRT3 knockout mice on a high-fat diet exhibited accelerated obesity, hyperlipidemia, and insulin resistance compared to the wild type mice [140]. In β-cells, SIRT3 plays a key role by regulating insulin secretion and modulates hepatic lipid metabolism via 5-hydroxytryptamine in a high-fat diet. In a mechanism standpoint, SIRT3 enhances fatty acid oxidation by deacetylating key enzymes including long-chain acyl-CoA dehydrogenase and carnitine palmitoyltransferase 2. Impaired SIRT3 function results in the accumulation of long-chain acylcarnitine, which is consistent with the elevated plasma long-chain acylcarnitine levels in patients with obesity [141]. In addition, an in vivo study investigating the effects of aerobic exercise on obesity showed that elevated SIRT3 expression contributes to weight improvement by activating pathways that regulate mitochondrial homeostasis [142]. These findings suggest that activating SIRT3 in adipose tissues offers a potential strategy to mitigate metabolic diseases linked to obesity.

SIRT6 has emerged as a protective regulator against diet-induced obesity and metabolic syndrome. In high-fat diets models, transgenic mice overexpressing SIRT6 accumulated markedly less visceral fat, triglycerides, and LDL-cholesterol compared to wild-type controls and displayed improved lipid metabolism in adipose tissue. Gene expression profiling in adipose tissue showed that the protective effects of SIRT6 overexpression are associated with the reduced expression of a selective set of PPAR responsive genes and lipid storage related genes such as angiopoietin-like protein 4, diacylglycerol acyltransferase, and adipocyte fatty acid-binding protein. These findings indicate that SIRT6 functions as a key regulator of adipose tissue homeostasis and protects against obesity-related metabolic complications [143]. In addition, SIRT6 in the hypothalamus is a key regulator of microglial activity and energy homeostasis. High-fat diets suppress SIRT6 expression in hypothalamic microglia, and microglia-specific SIRT6 knockout aggravates neuroinflammation and metabolic dysfunction, leading to hyperphagia, reduced energy expenditure, and insulin and leptin resistance. Mechanistically, SIRT6 deacetylates and stabilizes NRF2, upregulating antioxidant genes and protecting microglia from reactive oxygen species. Consistently, SIRT6 overexpression in BV2 cells protects against acid induced inflammation, whereas pharmacological inhibition of NRF2 negates these effects [144]. Collectively, these studies demonstrate that SIRT6 acts as a protective regulator in both peripheral adipose tissue and central hypothalamic microglia, highlighting its potential as a therapeutic target for obesity and obesity-related metabolic dysfunction.

### 4.3. Sirtuins and Osteoporosis

Bone is a dynamic tissue that continually remodels in response to functional demands through the coordinated activity of osteoblasts and osteoclasts. Disruption of this balance impairs bone formation and resorption, which leads to bone diseases such as osteoporosis. Growing evidence suggests that impaired cellular homeostasis caused by mitochondrial dysfunction, oxidative stress, and inflammation contributes to the onset and progression of osteoporosis [145]. Modulating sirtuin expression or activity was proposed as a potential therapeutic strategy for preventing and treating osteoporosis. SIRT1 knockout mouse embryos and newborns pups showed increased lethality compared to wild-type animals and developed abnormalities in the retina, bones, and heart. Specifically, the mineralization of cranial, vertebral, and digits bones was markedly delayed, suggesting that SIRT1 is essential for bone growth and remodeling. SIRT1 contributes to bone remodeling through hormones and endocrine signaling pathways. For example, estrogen enhances SIRT1 expression and ovariectomy diminishes it [139,146]. Transgenic mice overexpressing SIRT1 had 40–50% higher bone mass than the control group at 2.5 years, along with evidence of healthier aging. Overexpression of SIRT1 in mesenchymal lineage cells led to increased bone volume in both young and aged mice, which was attributed to elevated osteoblast and reduced osteoclast numbers [146]. Taken together, this suggests that activation of SIRT1 could be a new option for osteoporosis therapy.

Elevated hepatic SIRT2 promotes osteoporosis by increasing the release of leucine-rich α-2-glycoprotein 1 in extracellular vesicles, which subsequently suppresses osteoclast differentiation in bone marrow cells [147]. SIRT2 knockout rats showed higher bone volume fraction and trabecular density than wild-type rats at 36 weeks. Pharmacological inhibition of SIRT2 with AGK2 suppressed osteoclast differentiation [148]. However, further studies are needed to clarify the mechanisms by which SIRT2 regulates osteoclast differentiation and function, as well as its contribution to age-related bone loss.

SIRT3 maintains bone homeostasis through the regulation of oxidative stress, mitochondrial dynamics, and energy metabolism in bone cells. This regulation supports osteoblast differentiation and promotes bone formation. In mouse models, SIRT3 overexpression alleviated age-induced senescence and promoted the osteogenic differentiation of bone marrow mesenchymal stem cells, thereby preventing osteoporosis through the activation of mitophagy [149]. SIRT3 also enhances mitochondrial function in osteoclasts, thereby facilitating bone resorption. However, SIRT3 deletion or pharmacological inhibition in aged and estrogen-deficient mice reduced osteoclast activity and protected against bone loss [150]. SIRT3 might exhibit context-dependent roles in skeletal remodeling, requiring more research before therapeutics can be developed.

### 4.4. Sirtuin Activators for Treating Metabolic Diseases

Activators of sirtuins are currently used for the treatment of metabolic disorders. Metformin, a widely prescribed oral anti-diabetic drug for more than 60 years, remains the primary therapeutic option for type 2 diabetes [151]. Metformin activates SIRT1 directly, resulting in an increased deacetylation of downstream targets and the promotion of mitochondrial biogenesis and improvement of insulin sensitivity (Table 4, [152]). Metformin also indirectly modulates SIRT1 activity via AMPK-mediated activation. The activation of AMPK by metformin is mediated through inhibition of the mitochondrial respiratory chain complex I, which leads an increase in intracellular NAD^+^ that subsequently increases SIRT1 activity [153,154]. A randomized controlled trial on metformin for diabetes prevention enrolled 1078 participants from 27 U.S medical centers, assigning them to metformin (850 mg twice daily) or placebo. After 3 years, diabetes incidence was reduced by 31% in the metformin group compared with the placebo group [155]. In addition, metformin increases bone health and reduces bone loss in ovariectomized mice through suppressing autophagy in osteoclast precursors and protecting osteoblasts from H_2_O_2_-induced apoptosis. This protective effect is mediated through activation of the PI3K/AKT pathway, which enhances SIRT3 expression [156]. This suggests that activating sirtuins may be a therapeutic strategy to treat metabolic disorders.

Resveratrol exhibits a wide range of protective effects, including increasing glucose metabolism, and improving insulin sensitivity (Table 4). Resveratrol activates SIRT1 to improve mitochondrial function and shield against metabolic conditions. Mice treated with resveratrol showed a reduced risk of obesity and insulin resistance when exposed to a high fat diet [157,158]. Resveratrol has also been shown to promote mitochondrial biogenesis by blocking phosphodiesterase, which raises cAMP levels. Under these conditions, NAD^+^ levels become elevated, which may underlie SIRT1 activation by resveratrol [159]. Sirtuin activators are candidates for treating or possibly preventing metabolic disorders in high-risk patients.

The SIRT1 activator SRT2104 has been extensively studied in both preclinical and clinical trials for diabetic vascular complications (Table 4). Diabetic vascular complications are common and serious chronic conditions associated with diabetes. A total of 50–80% of diabetic patients develop cardiovascular issues, which account for approximately 70% of diabetes-related deaths. In animal studies, SRT2104 enhanced insulin sensitivity and regulated blood glucose levels more effectively. Elevated blood glucose suppresses SIRT1 expression and promotes vascular cell senescence, contributing to the progression of vascular complications [160]. Thus, SIRT1 activation could be a potential therapeutic strategy. In a preclinical study, diabetic mice had greater aortic reactivity, increased oxidative and inflammatory markers, elevated acetylation of p53, and diminished levels of SIRT1. SRT2104 treatment elevated aortic SIRT1 levels by 3.79-fold and significantly improved endothelial function. In addition, SRT2104 increased SIRT1 expression and reduced p53 acetylation, oxidative stress, and inflammation in high glucose treated endothelial cells. SRT2104 provided no additional benefit when p53 was knocked down [160,161]. These findings indicate that SRT2104 exerts its vascular benefits primarily through p53 deacetylation. SRT2104 enhanced renal SIRT1 activity and promoted p53 deacetylation in wild-type mice, resulting in the activation of the Nrf2 antioxidant pathway. This activation conferred protection against diabetes-induced renal damage, including oxidative stress, inflammation, fibrosis, glomerular remodeling, and proteinuria. Thus, SRT2104 may be useful as a therapy to delay the development of diabetic nephropathy [160,162]. A phase II, randomized, double-blind, placebo-controlled clinical trial (NCT00937326) was carried out to evaluate the safety and pharmacokinetics of multiple doses of SRT2104 in patients with type 2 diabetes. Over 28 days, participants received SRT2104, resulting in improved lipid profiles, including reduced LDL cholesterol and triglycerides. However, no significant changes were observed in blood glucose or insulin sensitivity, indicating limited effects on glycemic control [160,163]. The impact of SRT2104 on insulin sensitivity should be further explored with larger cohorts for a prolonged time.

**Table 4:** Summary table of SIRT1 activators with potential to treat metabolic diseases.

Compound	Mechanism of Action	Findings	REFs
Metformin	Directly activates SIRT1, promotes mitochondrial biogenesis, and improves insulin sensitivity. Indirect activation of SIRT1 via AMPK activation and ↑ NAD^+^ levels.	Used as a first-line therapy for type 2 diabetes	[152,153,154]
Resveratrol	Blocks PDE, ↑ cAMP levels ↑ calcium, ↑ NAD^+^ levels, thereby activating SIRT1.	In mice, decreased the risk of obesity and insulin resistance.	[157,158,159]
SRT2104	↑ SIRT1 expression, and reduces p53 acetylation, oxidative stress, and inflammation	In animal studies, SRT2104 enhanced insulin sensitivity and regulated blood glucose levels effectively. Phase II trial: 28 days improved lipid profile (↓ LDL and triglycerides), but no significant change in blood glucose or insulin sensitivity	[160,161,163]

↑ Increased level or activity; ↓ Decreased level or activity.

## 5. Sirtuins Role in Neurodegenerative Diseases and as Possible Therapeutic Targets

Neurodegenerative disorders are linked to a gradual and irreversible death of neurons in the brain, leading to a progressive loss of motor, physiological, and cognitive abilities that impair speech, breathing, and memory. The development of neurodegenerative diseases, such as Alzheimer’s disease (AD), Huntington’s disease (HD), and Parkinson’s disease (PD), are attributed to oxidative stress and mitochondrial dysfunction. Next, we discuss current knowledge on the role of sirtuins in these diseases and explore their possibilities as therapeutic targets.

### 5.1. Sirtuins as Possible Contributors to PD Development

PD is a progressive, degenerative condition that affects the nervous system, which leads to neuronal death and dopamine deficiency. It primarily affects the motor system, and may also cause tremors, stiffness, slowness of movement, difficulty walking, and, in rare situations, issues with cognition and behavior [164]. Although the exact cause of PD remains largely unknown, there is evidence suggesting that mitochondrial dysfunction, α-synuclein aggregation, oxidative stress, inflammation, and autophagy are factors in disease development [165]. In addition, there is growing evidence that PD is influenced by mitochondrial sirtuins. SIRT3 reduces oxidative stress and enhances the stability of the electron transport chain, thereby decreasing the impact of subcellular stress on mitochondria [58]. Acetylating manganese superoxide dismutase (MnSOD) at lysine 68 reduces its activity and results in a decreased ability to scavenge ROS. SIRT3-mediated deacetylation of MnSOD leads to its activation, thereby augmenting the detoxification of ROS [166]. In addition, SIRT3 increases the expression of MnSOD indirectly through the control of transcription factors [167]. Higher levels of MnSOD were detected in the substantia nigra and the frontal and motor cortex of PD patients [168]. In two separate clinical trials, MnSOD mRNA levels were found to be higher in whole-blood samples from PD patients compared to healthy controls. This suggests that MnSOD mRNA levels could serve as a potential blood biomarker for diagnosing PD [168,169]. Modulating both the activity and expression of MnSOD by activating SIRT3 is a possible therapeutic strategy for slowing PD progression. The use of the agonist honokiol, which is derived from the magnolia tree, to activate SIRT3 protects against neurodegeneration in PD [170]. A recent study found that PD model mice were shielded from motor impairments and progressive damage to primary dopaminergic neurons when treated with honokiol [171,172].

The mechanism and role of SIRT4 in PD is not fully understood. There is suggestive evidence, based upon quantitative proteomics using a PD mouse model, that SIRT4 regulates >5000 proteins [173]. SIRT4 mostly impacts peroxisome proliferator-activated receptor (PPAR) signaling [174], and peroxisome pathways [173,175]. However, among the 25 molecular targets examined, SIRT4 only regulated fatty acid binding protein 4 (FABP4) in the PPAR signaling pathway [173,176]. FABP4 is a signaling molecule that alters glucose and lipid metabolism and may contribute to the development of PD. When SIRT4 was overexpressed, there was increased expression of FABP4 and PPARγ. This suggests that FABP4 might be a SIRT4 target, but further functional validation is required [173].

SIRT1 might protect against PD by modulating neuroinflammation, reducing oxidative stress, and decreasing the aggregation of α-synuclein [177]. SIRT1 overexpression in neuroblastoma cells decreases α-synuclein and toxin-induced cell death and represses NF-κB and PARP activity regardless of its deacetylase activity [178,179]. In addition, the post-mortem brain tissue of male and female PD patients showed downregulated levels of SIRT1 [171]. SIRT1 activity might protect against PD, and activation could be a potential therapeutic strategy.

### 5.2. Sirtuins Role in AD Development

AD is the most prevalent cause of dementia and is currently the seventh-leading cause of death in the United States [180]. Amyloid-β (Aβ) plaques and neuropil threads, which result from aggregated, hyperphosphorylated tau proteins within the brain, are the hallmarks of AD, which is characterized by progressive memory loss [181]. Sirtuins may be involved in the development of AD, as there is a correlation between reduced SIRT1 levels and increased tau protein and Aβ deposition in those who have the disease [182]. The overexpression of SIRT1 in animal models reduced the production of Aβ and Aβ plaque formation while deleting SIRT1 increased Aβ levels [183]. Another study using AD mouse models confirmed that increased SIRT1 expression resulted in reduced symptoms of cognitive impairment, which helped to preserve learning and memory function [184]. Together, this suggests that SIRT1 overexpression is neuroprotective. This could be a potential treatment strategy to manage neurodegeneration and cognitive impairment in patients with AD. Mechanistically, SIRT1 deacetylates the retinoic acid receptor β, which activates ADAM10 (α-secretase) transcription. ADAM10 is an enzyme that cleaves the β-amyloid precursor protein to produce the Aα peptide. This prevents the production and build-up of the Aβ peptide and protects against neurodegeneration [171,185]. As a result, treatment approaches have centered on preventing the aggregation of Aβ, and SIRT1 activation could be a potential strategy for regulating Aβ levels.

AD patients have lower levels of SIRT3 expression in their cerebral cortex [186], which is due to a reduction in SIRT3 mRNA levels [187,188]. These findings suggest that SIRT3 activation could prevent or delay the onset and progression of AD. Additionally, SIRT3 deacetylates p53, which controls the levels of NADH dehydrogenase in brain tissue. This regulation is needed to regulate mitochondrial OXPHOS and prevent AD-associated neuronal damage [188,189]. SIRT3 also deacetylates tau and lowers its levels, potentially slowing the progression of AD [188,190]. SIRT3 activates MnSOD through deacetylation, thereby enhancing its antioxidant activity. Reducing intracellular levels of ROS could be beneficial [188].

In healthy individuals and mice, SIRT6 expression levels are typically stable or elevated compared to those with AD. In brain samples from AD patients, SIRT6 expression levels were decreased [191]. Aβ42 is a primary component of plaques in AD and induces DNA damage [192,193]. Among the various pathological features of AD, DNA damage, particularly double-strand breaks, leads to the loss of neuronal function and memory [194,195]. Aβ42-induced DNA damage was prevented by SIRT6 overexpression in mouse hippocampal neurons. This can be explained by the role of SIRT6 in maintaining chromatin structure and facilitating the repair of DNA double-strand breaks [193,196]. SIRT6 protects against Aβ42-induced DNA damage, suggesting that activation could become a possible therapy.

### 5.3. Sirtuins Role in HD

HD is an autosomal dominant genetic disorder caused by a trinucleotide repeat expansion in the huntingtin gene, leading to an abnormally long polyglutamine tract in the N-terminal domain. The mutant huntingtin (mHtt) gene, particularly with a repeat expansion exceeding 35, leads to HD. This disease results in a progressive breakdown and death of neurons in specific regions of the brain. Patients with HD develop involuntary motor symptoms, along with deficits in cognition, emotion, behavior, and personality [197]. The exact mechanisms through which the mHtt protein leads to neuronal dysfunction and degeneration are not yet fully understood. However, mitochondrial dysfunction is thought to contribute to mHtt-induced neurotoxicity [198,199]. Cells expressing mHtt had decreased SIRT3 levels [200,201]. mHtt disrupts various cellular functions, including transcription, translation, DNA repair, mitochondrial function, and nucleocytoplasmic transport. mHtt also impairs mitochondrial biogenesis and respiration, which disrupts neuronal energy metabolism. Overexpressing SIRT3 enhances antioxidant activity in cells with mHtt, improving mitochondrial function [202]. The SIRT3 activator ε-viniferin enhanced anterograde mitochondrial transport in neurites, thereby promoting cell survival. SIRT3 knockout mice treated with 3-nitropropionic acid were more sensitive to the toxic effects compared to wild-type controls [200,203]. Activation of SIRT3 could lead to new and improved HD treatment.

Studies in various animal models have demonstrated that SIRT1 protects against mHtt toxicity; however, recent findings indicate that both activation and inhibition of specific SIRT1 pathways be neuroprotective in HD [204]. The deacetylase activity of SIRT1 is essential for its neuroprotective effects in HD. mHtt binds to SIRT1 and suppresses its deacetylase function, leading to increased acetylation of its targets, which reduces cell survival [205]. SIRT1 overexpression mitigates neuropathology and upregulates brain-derived neurotrophic factor (BDNF) expression. Brain-specific SIRT1 deletion leads to increased severity of neurodegeneration in HD mice [206,207]. SIRT1-mediated neuroprotection depends on the CREB-regulated transcription coactivator 1 (TORC1), a brain-specific enhancer of CREB function. Under physiological conditions, SIRT1 activates TORC1 by deacetylating it, which promotes its dephosphorylation, and enhances its interaction with CREB. mHTT disrupts the interaction between TORC1 and CREB, resulting in decreased BDNF transcription [177,208]. The catalytic activity of SIRT1 is inhibited by nicotinamide, which binds to a conserved region in the active site and promotes a base exchange reaction over deacetylation. Nicotinamide enhances motor function and increases BDNF expression. This could be due to raising NAD^+^ levels rather than direct SIRT1 inhibition [207]. Nicotinamide serves as a precursor for NAD^+^ biosynthesis and is thought to enhance cellular energy production by increasing NAD^+^ availability [209]. It is possible that nicotinamide may increase NAD^+^ levels and activate SIRT1 in mice, implying that its benefits in HD are due to SIRT1 activation rather than inhibition [210]. Further investigation is needed to tease apart these possibilities.

### 5.4. Sirtuin Activators for Treating Neurodegenerative Diseases

**Figure 9:**
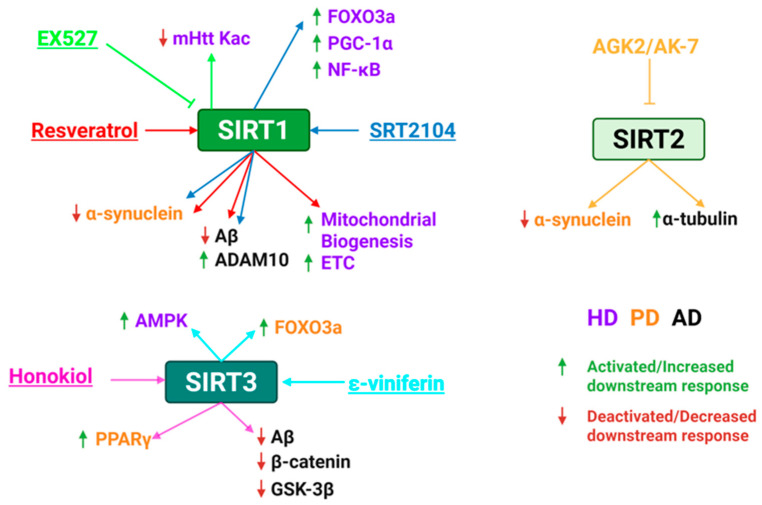
Summary of molecular effects of sirtuin activation or inhibition in neurodegenerative disorders by sirtuin modulators. SIRT1–3 are the targets of activators (resveratrol, SRT2104, honokiol, ε-viniferin) or inhibitors (EX527, AGK2, AK-7). Colored arrows match the modulator and depict the relevant downstream effect of sirtuin inhibition in HD (purple), PD (orange), or AD (black). Green arrows represent activation or increase in response, while red arrows represent deactivation or decrease in response. ETC, election transport chain. Created in BioRender. Carabetta, V. (2025) https://BioRender.com/y6fwujs.

Resveratrol activates SIRT1 and is neuroprotective in PD animal models by promoting autophagic degradation of α-synuclein and preserving dopaminergic neuron integrity (Figure 9 and Table 5). Although clinical studies are lacking, resveratrol has shown therapeutic potential in PD animal models. Resveratrol treatment improved motor and cognitive function in PD mice in a dose-dependent manner, likely through suppressing α-synuclein aggregation, lowering total and oligomeric α-synuclein, and reducing neuroinflammatory and oxidative damage [211]. In another study, resveratrol was orally administered for 10 weeks to assess its neuroprotective effects in a rat model of PD. The neuroprotective effect of resveratrol was linked to its ability to suppress inflammatory processes [212,213]. Clinical trials are needed to assess the dosing, safety, and therapeutic benefits in PD patients.

**Table 5:** Summary table of SIRT activators as a therapy for neurodegenerative diseases.

Compound	Target	Disease	Mechanism of Action	Findings	REFs
Resveratrol	SIRT1	PD	Promotes autophagic degeneration of α-synuclein, reduces neuroinflammation and oxidative damage	Improved motor and cognitive function in mice.	[211,212,213]
		AD	Increases ADAM10, reduces Aβ levels, modulates inflammation	Neuroprotective in AD models. Phase II trials show its safe, greater brain volume loss, reduced Aβ accumulation in the brain.	[214,215]
		HD	Enhances mitochondrial biogenesis and electron transport chain activity	Improved motor coordination and mitochondrial gene expression mouse models; Clinical trial completed but unpublished.	[216,217]
Honokiol	SIRT3	PD	Restores motor function, prevents dopaminergic neuron loss, reduces oxidative stress, etc.	Neuroprotective in PD models with improved behavioral outcomes.	[218]
		AD	Improves mitochondrial ATP production, reduces ROS, enhances mitophagy and neuronal survival	Improved memory and spatial learning in mouse models; reduced Aβ aggregation, oxidative stress, and NF-κB.	[171,218,219,220]
SRT2104	SIRT1	PD	Restores autophagy, reduces dopaminergic neuron loss	Improved coordination and motor function in PD mice; restored autophagy	[160]
		AD	Protects cerebrovascular endothelial cells from Aβ-induced stress, reduces endothelial dysfunction	Improve endothelial viability; reduce detrimental cognitive effects	[160]
		HD	Improves mitochondrial function and autophagy	Improve motor coordination, reduced brain atrophy, prolonged survival in HD mice	[221,222]
ɛ-Viniferin	SIRT3	PD	Enhances FOXO3 deacetylation/nuclear translocation, boosts ATP, reduces ROS	Reduced mitochondrial depolarization and apoptosis.	[223]
		HD	Stimulates AMPK, promotes mitochondrial biogenesis	Reduced ROS, prevented mitochondrial dysfunction in HD models	[203]

In AD models, resveratrol results in increased ADAM10 expression and reduced Aβ levels (Figure 9 and Table 5). In a phase II clinical trial involving patients with mild to moderate AD, oral doses of resveratrol administered once daily were safe and well-tolerated, with mild adverse effects. The cerebrospinal fluid and plasma Aβ40 levels declined significantly more in the placebo group compared to the resveratrol-treated group. However, resveratrol treatment was associated with greater brain volume loss relative to the placebo. [214]. Additionally, resveratrol and its main metabolites crossed the blood–brain barrier and exerted effects in the CNS. Another trial found that patients receiving resveratrol for 52 weeks showed lower levels of MMP-9, an inflammation-related marker associated with AD, compared to the placebo group. Additionally, resveratrol-treated patients experienced a smaller decline in CSF, leading to reduced Aβ accumulation in the brain [215]. Additional clinical trials are needed to firmly establish the potential benefit of resveratrol treatment in AD.

Multiple studies using HD models have also demonstrated the neuroprotective potential of resveratrol (Figure 9 and Table 5). Resveratrol treatment over 28 days in mice resulted in better motor coordination and learning and activated mitochondrial electron transport gene expression by restoring membrane potential [216]. Additionally, oral gavage of resveratrol in HD transgenic mice protected peripheral tissues from mHtt-induced damage [217]. A clinical trial (NCT02336633) evaluating the effect of resveratrol on the rate of caudate volume loss in HD patients has been completed, but the findings from the study have not yet been published. Additional clinical trials are needed to examine the potential of resveratrol to reduce neurodegeneration in individuals with HD.

Honokiol is currently being investigated for the treatment of neurological disorders (Figure 9 and Table 5, [219]). In PD models, honokiol exerts neuroprotective effects by restoring motor and behavioral function, preventing dopaminergic neuron loss, reducing oxidative stress, and enhancing PPARγ expression [218]. Although there are no human clinical trials for treating PD yet, the safety profiles of honokiol are encouraging. In the early stages of AD, memory deficits were improved, and spatial learning and memory were promoted by therapeutic modulation of SIRT3 activity with honokiol in mouse models. Honokiol increased SIRT3 expression in mitochondria, improved ATP production, and decreased mitochondrial ROS production [171,219,224]. In vitro analysis demonstrated that honokiol significantly inhibited Aβ42 aggregation, reduced cholinesterase activity, iron(II) chelation, and antioxidant activity through free radical scavenging [225]. Hyperactivation of glycogen synthase kinase 3β (GSK-3β) and decreased β-catenin levels contribute to oxidative stress and neuronal death, which is involved in AD pathogenesis. Honokiol treatment reversed these effects, downregulating GSK-3β and upregulating β-catenin, demonstrating neuroprotective and antioxidant effects [218,220]. Honokiol improved neuronal function in AD models by activating mitophagy, reducing Aβ aggregation, apoptosis, and oxidative stress, and improving mitochondrial membrane potential [226]. Additionally, honokiol improved cognitive function by reducing oxidative stress, lowering acetylcholinesterase and NF-κB levels, decreasing Aβ plaques and tangles, and maintaining neuronal integrity [227]. Honokiol has not yet been studied in any registered or ongoing clinical trials for the treatment of AD in humans.

SRT2104 activates SIRT1 in the brain, triggering several neuroprotective mechanisms, lowering inflammation, preventing cell death, and enhancing mitochondrial function and autophagy (Figure 9 and Table 5). In mice, chronic dietary administration of SRT2104 led to detectable concentrations in the brain [221], meaning that it crosses the blood–brain barrier and could be used to treat neurological disorders. In a PD mouse model, SRT2104 restored autophagy activity in the midbrain, decreased the loss of dopamine-producing neurons, decreased α-synuclein aggregation, and improved coordination and motor function in the affected mice. SRT2104 improved the viability of cerebrovascular endothelial cells in a dose-dependent manner under Aβ-induced stress, providing protection against endothelial injury. It also reduced the detrimental effects of endothelial dysfunction on cognitive performance. These findings highlight the therapeutic potential of SRT2104 in AD, although further studies are needed to evaluate its safety, efficacy, and mechanisms of action [160]. Chronic administration of SRT2104 in HD mice improved motor coordination, decreased brain volume loss, and prolonged survival. This could be due to increased NF-κB, PCG-1α, and FOXO3a signaling, which reduce neuroinflammation and oxidative stress. The treatment also reduced brain atrophy, especially in the neocortex, a region critically impacted in HD [221]. Human trials conducted so far have primarily focused on safety and pharmacokinetics in healthy individuals and for conditions like psoriasis [222]. Large-scale clinical trials specifically for neurodegenerative diseases are not currently active.

Trans-(-)-ε-viniferin, commonly called ε-viniferin, is a bioactive stilbene compound and a dimer of resveratrol (Figure 9 and Table 5). It occurs naturally in several plant species, notably in grapes (*Vitis vinifera*). ε-Viniferin is an SIRT3 activator and demonstrates neuroprotective properties in the context of PD and HD. In a cell model of PD, ε-viniferin treatment led to an increase in SIRT3 expression, which in turn enhanced the deacetylation and nuclear translocation of FOXO3a. Additionally, ε-viniferin boosted ATP production and reduced the levels of ROS. It also mitigated mitochondrial depolarization and cell apoptosis while restoring the expression of proteins involved in maintaining mitochondrial homeostasis [223]. In cells expressing mHtt, ε-viniferin reduced ROS and prevented mitochondrial membrane potential loss. ε-Viniferin activates SIRT3, stimulates AMP-activated kinase (AMPK), and promotes mitochondrial biogenesis. Silencing SIRT3 significantly reduces ε-viniferin-induced activation of AMPK and weakens its neuroprotective effects [203]. More research is needed to fully understand the mechanism of action and evaluate safety and efficacy.

### 5.5. Sirtuin Inhibitors for Treating Neurodegenerative Diseases

Hyperactivation of SIRT1 leads to disrupted transcriptional regulation in neurons, which partial inhibition could counteract. In preclinical models, EX527 exhibited cytoprotective effects in cells expressing mHtt and was neuroprotective in primary rat neurons transfected with human mHtt (Figure 9 and Table 6). Additionally, EX527 increased the acetylation at specific sites in mHtt in cellular models, leading to increased macroautophagic degradation. In a *Drosophila* model of HD, EX527 exhibited neuroprotective properties and mimicked the phenotype associated with SIRT1 haploinsufficiency [228,229]. In a Phase I clinical trial involving healthy volunteers, EX527 was found to be safe and well-tolerated across all dosing levels. In a double-blind, placebo-controlled study involving HD patients, EX527 was well-tolerated with no reported adverse effects; however, it did not alter circulating levels of soluble huntingtin over the short treatment period [230,231]. A 12-week clinical trial involving EX527 was conducted in HD patients, but no data from the study has been published. While clinical trials support its safety, further long-term clinical trials are required to determine its clinical benefit.

**Table 6:** Summary table of sirtuin inhibitors as a therapy for neurodegenerative diseases.

Compound	Target	Disease	Mechanism of Action	Findings	REFs
EX527	SIRT1	HD	Increases acetylation of mHtt, enhances macroautophagic degradation of mutant protein, reduces toxicity	Phase I trials: safe and well-tolerated. Phase II HD trials: safe, but no effects on circulating levels of soluble Htt.	[228,229,230,231]
AGK2	SIRT2	HD	Protects against α-synuclein toxicity, reduces neuronal death, decreases sterol biosynthesis.	Limited BBB permeability, restricts in vivo efficacy.	[232,233]
		PD	Mitigates α-synuclein toxicity; protected dopaminergic neurons.		
AK-7	SIRT2	HD	Increases α-tubulin acetylation, reduces mHtt aggregates and degeneration, improves motor function.	Improved BBB penetration, no clinical trials	[234,235]
		PD	Protects substantia nigra dopaminergic neurons, reduces oxidative stress, and mitochondrial dysfunction.		

SIRT2 is expressed in the cortex, hippocampus, and spinal cord. AGK2 exhibits a neuroprotective effect by mitigating the damage caused by α-synuclein in dopaminergic neurons (Figure 9 and Table 6). In a preclinical study conducted, the inhibition of SIRT2 showed a neuroprotective effect in both cellular and invertebrate models of HD. AGK2 treatment in primary striatal neurons expressing mHtt led to reduced neuronal death and suppressed aggregation. mHtt fragments elevated sterol levels in neuronal cells, and inhibition of SIRT2 by AGK2 reduced them. Altering sterol biosynthesis at the transcriptional level mimicked the effects of SIRT2 inhibition, indicating that the metabolic changes triggered by SIRT2 inhibition are sufficient to lessen the toxicity of mHtt [232]. In a *Drosophila* model of PD, SIRT2 inhibition mitigated α-synuclein toxicity and prevented the death of dopaminergic neurons. In neuroglioma cells with elevated α-synuclein expression, AGK2 protected against α-synuclein-induced toxicity. AGK2 facilitates the formation of larger, protective inclusion bodies, which may sequester harmful α-synuclein aggregates and shield dopaminergic neurons from degeneration [233]. Although AGK2 is a potent and selective SIRT2 inhibitor with in vitro neuroprotective effects, its limited permeability across the blood–brain barrier restricts its efficacy in animal models and limits its potential to treat neurodegenerative disorders. AK-7 is a selective SIRT2 inhibitor, designed to address the limited CNS bioavailability seen with AGK2 (Figure 9 and Table 6). In a cell model of HD, AK-7 treatment showed increased acetylation of α-tubulin, decreased aggregated mHtt, enhanced motor function, reduced brain degeneration, and prolonged lifespan [234]. Moreover, in both cellular and animal models of PD, AK-7 treatment significantly protected dopaminergic neurons in the substantia nigra, reduced motor deficits, and mitigated oxidative stress and mitochondrial dysfunction [235]. 

## 6. Challenges and Future Directions

Although extensive progress has been made elucidating the roles of sirtuins in cancer, metabolic, and neurodegenerative diseases, major questions remain regarding their isoform-specific functions, context dependent effects, and translational safety. A major challenge in translating sirtuin-targeted therapies lies in achieving isoform selectivity. Although compounds such as resveratrol, SRT2104, and EX527 were promising in preclinical studies, their limited selectivity and toxicity limit their clinical application. The dose-dependent duality of sirtuin activity, where both excess activation and inhibition can be detrimental, emphasizes the need for therapeutic strategies that balance the modulation of sirtuin activity rather than broad-spectrum activation. In cancer, the clinical application of sirtuin modulators is highly context-dependent, requiring tailored clinical strategies that consider the cancer type, the tumor microenvironment, and the specific sirtuin expressed in the target tissue. Future studies should focus on elucidating the tissue specific and context dependent roles of these sirtuins. Achieving selective and efficient drug delivery to tumor cells is another obstacle. Systemic administration often leads to off-target effects, reduced therapeutic efficacy, and increasing toxicity to normal tissues. To address these limitations, emerging strategies such as nanoparticle-based delivery systems, tumor-targeted prodrugs, and ligand-guided formulations are being explored. These delivery systems should enhance tumor specificity, improve drug accumulation within the tumor microenvironment, and minimize undesired exposure to normal tissues [236,237].

Pharmacokinetic limitations, including low bioavailability, rapid metabolic clearance, and poor blood–brain barrier penetration of sirtuin modulators further limits their clinical applications, especially for neurodegenerative diseases like AD, PD, and HD [238]. The development of sirtuin-targeted therapies should focus on designing modulators with improved bioavailability, strong efficacy, reduced toxicity, and enhanced selectivity. This can possibly be achieved with in silico approaches and by taking advantage of artificial intelligence platforms.

Drug discovery relies heavily on the structural and mechanistic understanding of the conserved NAD^+^-dependent catalytic core and its variable surrounding domains. The catalytic core consists of a large Rossmann-fold responsible for NAD^+^ binding and a smaller zinc-binding domain that contributes to substrate recognition and structural stability. The flexible cofactor-binding loop and substrate-binding pocket are the regions exploited for selectivity and potency optimization. The pharmacophore models of sirtuin modulators typically include features that mimic the NAD^+^, hydrogen-bond donors or acceptors that interact with conserved residues, hydrophobic/aromatic groups that occupy acyl channel, or the pocket involved in nicotinamide exchange. For example, EX-527 binds in the conserved nicotinamide pocket, blocking NAD^+^ turnover [112], whereas activators such as resveratrol activate SIRT1 by binding to an allosteric site in its N-terminal regulatory region, inducing a conformational change that enhances the activity of the enzyme [183]. Targeting isoform-specific loops and non-catalytic extensions has become a common strategy to improve selectivity [239]. Understanding the relationship between domain architectures, catalytic mechanisms, and ligand binding may enable the rational design and optimization of sirtuin modulators with improved potency, selectivity, and pharmacokinetic profiles.

Improvement of systemic and tissue specific NAD^+^ bioavailability to regulate sirtuin activity might be another avenue to explore. Endogenous NAD^+^ levels decline with age in numerous pathological states, including cancer, metabolic, and neurodegenerative diseases, leading to impaired sirtuin regulation [240]. Current strategies to boost NAD^+^ levels, such as nicotinic acid, nicotinamide riboside, and nicotinamide mononucleotide, have been promising; however, challenges remain regarding bioavailability, long-term safety, stability, and tissue distribution. Many precursors of NAD^+^ undergo extensive metabolism and may not effectively raise intracellular NAD^+^ levels in all organs [241]. Development of NAD^+^ precursors and sirtuins modulators with improved pharmacokinetics and tissue selectivity could result in novel treatment options. CRISPR-mediated gene activation (CRISPRa) represents another approach that could be used to activate sirtuins. A recent CRISPRa screen identified guide RNAs (gRNAs) that strongly activated the expression of SIRT1. This suggests that CRISPRa screening platforms can be used to identify gRNAs that activate multiple therapeutically significant genes in a rapid and streamlined manner [242]. Future studies should utilize this technique to activate additional sirtuin isoforms and fully evaluate the gRNAs’ effects in certain disease states, as they may offer therapeutic benefits.

Finally, while SIRT1, SIRT2, SIRT3, and SIRT6 have been extensively characterized in various disease states, the pathological functions of SIRT4, SIRT5, and SIRT7 remain underexplored. Their potential involvement in metabolic reprogramming, mitochondrial quality control, and epigenetic regulation under disease contexts warrants further investigations. Understanding these underexplored sirtuins may reveal novel therapeutic avenues and refine the broader understanding of sirtuin biology in human health and disease.

## 7. Conclusions

A wide range of human diseases including cancer, diabetes, and neurodegenerative diseases can be potentially treated by modulating sirtuin activity. Small-molecule modulators have shown promise in preclinical models, highlighting their potential as therapeutic agents across diverse clinical applications. However, many sirtuins function as either tumor suppressors or oncogenes depending on the cancer type, disease stage, and cellular context, which complicates their clinical translation. Both activators and inhibitors of sirtuins could serve as anti-cancer agents due to their ability to regulate apoptosis, cellular metabolism, angiogenesis, and DNA repairs. Furthermore, sirtuin modulators may enhance tumor sensitivity to chemotherapy and radiation, suggesting their utility as adjuvants in combination therapies. SIRT1, SIRT3, and SIRT6 activation may be a new treatment strategy for various metabolic diseases including diabetes, obesity, and osteoporosis. Compounds such as metformin, resveratrol, and SRT2104 have demonstrated encouraging preclinical efficacy, although long-term clinical trials are required to validate their safety and therapeutic potential. Sirtuin activators can also be neuroprotective by improving mitochondrial function and lowering oxidative stress and neuroinflammation. Collectively, modulating sirtuin activity may be a powerful mechanism to treat or manage a wide array of human diseases.

## Figures and Tables

**Table 1:** Summary of localization, enzymatic activity, and functional roles of sirtuins.

Isoforms	Localization	Enzymatic Activity	Functional Roles
SIRT1	Nucleus	Strong deacetylase activity; regulates transcription factors	Aging, metabolism, inflammation
SIRT2	Cytoplasm	Strong deacetylase activity; targets, histones, cell-cycle regulators	Cell cycle control
SIRT3	Mitochondria, nucleus	Strong deacetylase activity; regulates metabolic enzymes	Mitochondrial homeostasis
SIRT4	Mitochondria	Weak deacetylase activity, ADP-ribosyltransferase, lipoamidase, and deacylase activities	Regulates metabolism, insulin secretion
SIRT5	Mitochondria	Low deacetylase activity; potent desuccinylase, demalonylase, and deglutarylase activity	Fatty acid oxidation, regulation of glycolysis, amino acid breakdown, and cellular respiration
SIRT6	Nucleus	Moderate deacetylases activity, strong ADP-ribosyltransferase, defatty-acylase activity	DNA repair, genomic stability, metabolism, inflammation, aging
SIRT7	Nucleus	Moderate deacetylase activity; regulates Pol I transcription and rRNA synthesis	Ribosome biogenesis, stress resistance

## Data Availability

No new data were created or analyzed in this study. Data sharing is not applicable to this article.

## Abbreviations

The following abbreviations are used in this manuscript:

AD	Alzheimer’s disease
PD	Parkinson’s disease
HD	Huntington’s disease
PTMs	Post-translational modifications
KATs	Lysine acetyltransferases
KDACs	Lysine deacetylases
HDACs	Histone deacetylases
NAM	Nicotinamide
SIRTs	Sirtuins
FOXO	Forkhead box O
NF-kB	Nuclear factor kappa-light-chain-enhancer of activated B cells
PGC-1α	Peroxisome proliferator-activated receptor gamma coactivator 1-alpha
EMT	Epithelial-mesenchymal transition
ROS	Reactive oxygen species
HIF1α	Hypoxia-inducible factor 1α
PDHA1	Pyruvate dehydrogenase complex E1α subunit
LDHA	Lactate dehydrogenase
TCA	Tricarboxylic acid
LAMP2	Lysosomal-associated membrane protein 2
MnSOD	Manganese superoxide dismutase
IDH2	Isocitrate dehydrogenase 2
PPAR	Proliferator-activated receptor
FABP4	Fatty acid binding protein 4
Aβ	Amyloid-β
mHtt	Mutant huntingtin
BDNF	Brain-derived neurotrophic factor
TORC1	Transcription coactivator 1
GKRP	Glucokinase regulatory protein
GCK	Glucokinase
OXPHOS	Oxidative phosphorylation
Drp1	Dynamin-related protein 1
